# A possible new cardiac heterogeneity as an arrhythmogenic driver

**DOI:** 10.1038/s41598-023-33438-y

**Published:** 2023-05-10

**Authors:** A. Rabinovitch, R. Rabinovitch, Y. Biton, D. Braunstein, R. Thieberger

**Affiliations:** 1 Physics Department, Ben-Gurion University, Beer-Sheva, Israel; 2 Makif YudAlef, Rishon Lezion, Israel; 3 Physics Department, Sami Shamoon College of Engineering, Beer-Sheva, Israel

**Keywords:** Cardiac device therapy, Cardiology, Cardiovascular diseases

## Abstract

Atrial fibrillation (AF) is the commonest cardiac arrhythmia, affecting 3 million people in the USA and 8 million in the EU (according to the European Society of Cardiology). So, why is it that even with the best medical care, around a third of the patients are treatment resistant. Extensive research of its etiology showed that AF and its mechanisms are still debatable. Some of the AF origins are ascribed to functional and ionic heterogeneities of the heart tissue and possibly to additional triggering agents. But, have all AF origins been detected? Are all accepted origins, in fact, arrhythmogenic? In order to study these questions and specifically to check our new idea of intermittency as an arrhythmogenesis agent, we chose to employ a mathematical model which was as simple as possible, but which could still be used to observe the *basic network* processes of AF development. At this point we were not interested in the detailed ionic propagations nor in the actual shapes of the induced action potentials (APs) during the AF outbreaks. The model was checked by its ability to exactly recapture the basic AF developmental stages known from experimental cardiac observations and from more elaborate mathematical models. We use a simple cellular automata 2D mathematical model of N × N matrices to elucidate the field processes leading to AF in a tissue riddled with randomly distributed heterogeneities of different types, under sinus node operation, simulated by an initial line of briefly stimulated cells inducing a propagating wave, and with or without an additional active ectopic action potential pulse, in turn simulated by a transitory operation of a specific cell. Arrhythmogenic contributions, of three different types of local heterogeneities in myocytes and their collaborations, in inducing AF are examined. These are: a heterogeneity created by diffuse fibrosis, a heterogeneity created by myocytes having different refractory periods, and a new heterogeneity type, created by intermittent operation of some myocytes. The developmental stages (target waves and spirals) and the different probabilities of AF occurring under each condition, are shown. This model was established as being capable of reproducing the known AF origins and their basic development stages, and in addition has shown: (1) That diffuse fibrosis on its own is not arrhythmogenic but in combination with other arrhythmogenic agents it can either enhance or limit AF. (2) In general, combinations of heterogeneities can act synergistically, and, most importantly, (3) The new type of intermittency heterogeneity proves to be extremely arrhythmogenic. Both the intermittency risk and the fibrosis role in AF generation were established. Knowledge of the character of these arrhythmogenesis agents can be of real importance in AF treatment.

## Introduction

Atrial fibrillation (AF) is the commonest arrhythmia worldwide (^1^ and references therein). Its incidence increases in the elderly (estimated lifetime risk, 22%–26%), and it is a source of morbidity and even mortality. Treatments include drugs, electric shocks and different types of ablations. Still, despite the vast effort that has been carried out in investigating AF pathogenesis, predisposing actors, and genetics, at least 1/3 of patients are resistant to treatment^1^. This partial failure raises two major problems: 1. are all accepted origins, in fact, arrhythmogenic? 2. have all AF origins been detected?

AF mechanisms are still controversial. They are attributed to: 1. cellular processes^2^, such as electrical remodeling of ion channels, gap junctions and abnormal Ca^2^^ +^ management in the sarcoplasmic reticulum. 2. Tissue changes in both myocardium and interstitium^3^. Specifically, the role of fibrosis is considered e.g., in^4^. 3. Inflammation and autonomic nervous changes^5^. 4. Myocyte-fibroblast coupling^6^. But the direct source of AF is usually considered to be drivers, located at abnormal places or positions in a heterogeneous atrial tissue which are activated by a specific external trigger^3^.

A debate still exists about these AF-prompting drivers. The two main ones are a spiral (rotor), or a double spiral, and a focal pulse source, termed focal breakthrough. A spiral is a functional reentry assumed to originate when an AP wavefront interacts with some form of barrier, such as a unidirectional block^7,8^. It can lead to heart tachycardia, or by encountering other obstacles like scars or blood vessels, can also break up into several sub-spirals and cause fibrillation. A double spiral is denoted in cardiology as a "figure of eight". A focal driver can arise by a breakthrough from within the cardiac tissue. Its action is characterized by spreading target waves (called also focal waves) emerging from a single point-like source and can induce AF while its spreading-wave function lasts.

Heart tissue response to an action potential (AP) propagation is not homogeneous. There are differences between ventricles and atria^9^ and also within each of their segments^10^.

More worrisome heterogeneities, which can lead to heart malfunctions^11^ are the subject matter of this work. Heterogeneities included in this study are local differences in conduction and in the refractory period of specific cells or of cell-groups, with respect to their general surroundings. These can be caused by obesity^12^, from lesions initiated by infarct^13^ or by tissue fibrosis^14^. We analyze each type of heterogeneity in order to evaluate its relation to atrial fibrillation.

Considering conditions for AF appearance, it is accepted that "substrates", such as heterogeneities, are the major *necessary* conditions for such appearance^11,15^. However, these conditions are not always *sufficient ones* under sinus node regular pacing. Experimental and theoretical studies have found that three different circumstances are prevalent. Some heterogeneity-types are sufficient by themselves, even under sinus node pacing to cause AF. For other heterogeneity-types there must exist an additional element, a trigger, which can be supplied by an ectopic AP pulse, to achieve an outbreak of AF; and for yet other heterogeneity-types these conditions, even with the trigger present, are insufficient to induce AF.

An ectopic electric pulse can originate from after depolarization^16^, either early (EAD) or delayed (DAD), or from a different origin, emanating from an ectopic source. Such a source situated at the veins was noticed in 1998 by the seminal work of Haissaguerre et al^17^ as a driver of AF and has since been used as the motivation of pulmonary vein isolation (PVI) in ablation procedures.

Particularly, the arrhythmogenesis of heart *fibrosis* is controversial. In our previous paper^18^ we showed that the mere existence of distributed (diffuse) or even localized fibrosis in the tissue when the heart's function is carried out by the regular sinus rhythm, does not lead to an arrhythmic result. Following experimental results^19–22^, some authors claim that atrial fibrosis is the main origin of AF while others maintain that it only supports other sources, and it is well known that people with atrial fibrosis may not suffer from AF at all. Fibrosis can be categorized in several ways (^23^ and see the very illuminating figures in this reference). One way is to differentiate between replacing and interstitial fibrosis, i.e., between fibrous tissue that replaces damage myocytes and that appearing between myocytes. For replacing fibrosis, one can differentiate between fibrotic patches and diffuse or distributed fibrosis, where in interstitial fibrosis the fibrotic cells are randomly distributed among healthy myocytes. We consider here only diffuse fibrosis. Other types are subject matter for future study.

Some experimental^24^ and advanced mathematical models (See the review^25^) have attempted to identify the exact conditions needed to initiate atrial fibrillations. However, the *basic physical* processes, i.e., the *cellular network* processes and the exact encounters between the trigger and the substance, are still not completely understood. We believe that a better understanding of such processes could help in advancing new treatments of heart arrhythmia problems such as tachycardia, flutter and fibrillation.

Our aim is to use the basic mechanisms of AF to look for new drivers leading to the disorder. Using Albert Einstein's paradigm, (the simplest mathematical model that can still fulfill the task), we used a cellular automaton method. This system has several advantages^26^ over the customary models which are based on differential equations. It takes full account of the cellular character of the medium, can accurately weigh the neighboring cellular influence on a site and illustrate the cellular patterns of the phenomena.

An interesting more elaborate *cellular* mathematical model^27^ simulating cardiac alternans incorporates two different established mathematical models. Both models focus on the ionic changes during AP developments, where one model treats fast changes of calcium ions while the other is statistical in nature. Since no model can be a complete simulator of the actual cardiac function, and since we were interested only in the basic electrical behavior of the network and not in exploring the intricate ionic processes nor in finding the exact AP shapes, we chose, at this stage, to use the simpler statistical *cellular* automata model which provides a good representation of this behavior.

We analyze the effects of three conduction heterogeneities under a sinus node wave, with and without an additional ectopic AP pulse: 1. heterogeneity in the refractory period, used here in order to verify the validity of our model by checking that the results obtained from it are in agreement with known results from experiments and from more elaborate mathematical models. 2. Heterogeneity in diffuse fibrosis. Fibrosis of any nature is considered by some cardiologists to promote reentry formation leading to AF (see^4^ and references therein). Patchy fibrosis has been theoretically considered by us previously^18^ and shown not to induce AF on its own. Therefore, we use here only diffuse fibrosis, and try to find out its role in AF arrhythmogenesis and its assistance to other arrhythmogenic agents. 3. *A novel heterogeneity*, we would like to call an electric conduction *intermittent* heterogeneity (ECIH), one which is explained in detail in the results section. We also investigate combined heterogeneity types, e.g., heterogeneity in refractory period and fibrosis, to ascertain whether multi-heterogeneities aid or deter AF.

## The model

The model was described in detail in our previous paper^18^. Here we give a short outline of it with the additional characteristics of its present version.

We use a 2D square cellular automata matrix of *n* × *n* cells. Time is discrete and measured by generations (or time units).

Note that since a single heart cell can hardly induce a strong enough electric current for an AF trigger, a "cell" here means a small cellular tissue which is large enough to produce such a trigger.

Each cell at each generation can assume several characteristic *modes* labeled by digital numbers from 0 to 5 (and 9, see below). A cell, which is at mode 1, is operating, namely containing an action potential (AP). The label 0 denotes a "waiting" cell, a cell that can be excited (see below) into its operating mode. Labels 2, 3 etc. denote cells in their refractory period, namely cells which cannot be stimulation into becoming operational. The final number (up to 5) denotes the end of the refractory period.

Label-transitions from one generation to the next is done for all cells according to the following rules:

A cell labelled 1, is transferred to label 2 (if present in this trial); a cell labelled 2, passes over to label 3 (if present in this trial), 3 goes to 4 etc.; a cell with the final label of refractoriness for this trial, changes to 0 in the next generation (Thus, if this final-refractory-period label is 3, namely the refractory period's duration is 2 generations, an operating cell labelled 1 passes in the next generation to label 2, in the next generation to label 3 and to 0 in the next generation); a cell labelled 0, transfers to 1 in the following generation if one or more of its nearest neighbors was operative, i.e. labelled 1 at the present generation. A cell passing through the refractory period process is not influenced by its neighboring cells.

Distributed (diffuse) fibrosis is investigated by rendering, at the beginning of each trial, a specific percentage, *q*, of cells to be *inoperative*, labelled 9 (fibrotic). These are uniformly and randomly spread among all grid cells, excluding the first and last rows of the grid. Note that cells adjacent to a "fibrotic" cell may also be fibrotic, since there is no avoidance of fibrosis locations. Such scarce adjacent fibrosis cites are not considered "patchy".

At the start of each trial, all cells except those having a label 9 and those in the first row, are labelled 0. Sinus node operation is represented by a single propagating AP wave, a sinus node wave (SNW) which is initiated by stimulating all cells of the first row to turn operative (labelled 1) for a single time unit.

In this work, an additional triggering operation is supplemented by a *temporary ectopic source*, which is represented by a single cell turned operative (labelled 1) for a single generation. This cell is chosen either on the first row or on the first column of the 2D matrix and its operation is delayed with respect to the sinus wave by predetermined *m* time units. We did not investigate the origin of this ectopic pulse, but only explored its influence on the system and its AF creating capability. Such a pulse, whose appearance in the atria is called premature atrial complex (PAC), if operating by itself, is propagating in a homogeneous tissue as a spreading (so called "target") wave, and, again, no AF is initiated by it. If, on the other hand, it is close enough in time, in the so called "vulnerable window", to the sinus node wave, then, due to tissue heterogeneity, AF generation can develop.

We treat the arrhythmic influence by changing the heterogeneity-percentage in the different cases and the delay of the ectopic pulses with respect to the sinus waves.

**Figure 1:**
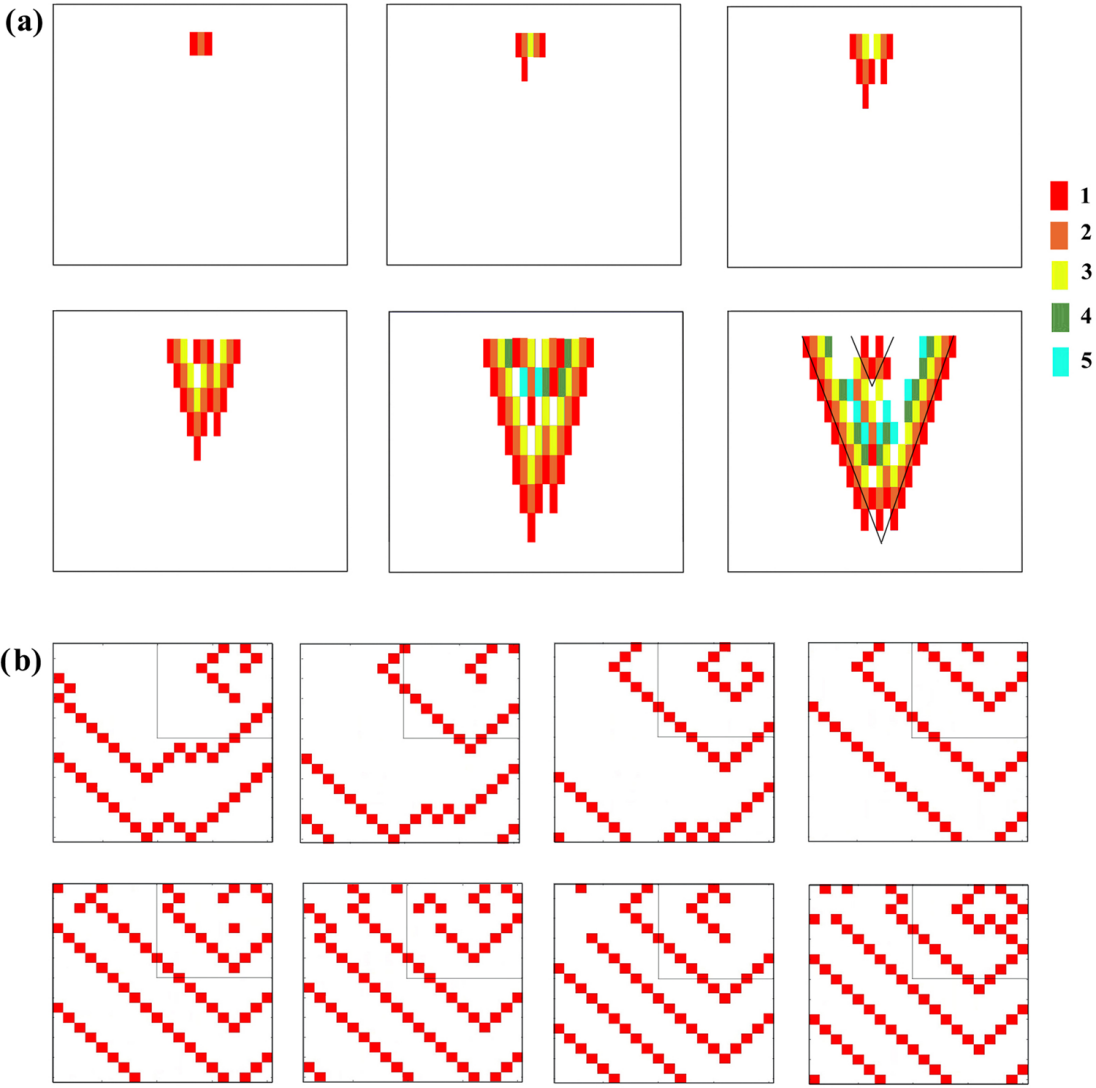
Refractory period (action potential duration APD) heterogeneity: A combination of an A-type (short refractory period, 1 → 2 → 3 → 0) and a B-type (long refractory period or normal 1 → 2 → 3 → 4 → 5 → 0) cells in a 2D matrix (20 × 20). Initial steps of the AF developments of the two types are shown: (**a**) Generation of a "focal ectopic" source. Several stages of the development are shown. Note the rectangular shapes of the outwards spreading waves. (**b**) Generation of a "spiral (rotor)" source. Several stages of the development are shown. In order to highlight the spiral development, only active cells (1's) are displayed (in red).

Recall that the sinus operation is represented here by a wave front of AP (of activations of cells) starting at the *first* row of *n* cells of the tissue (matrix) under consideration. AF is specified if the number of activations of cells at the *last* row, when only a sinus wave has been initiated, should be above *n*. When both the sinus wave and an additional pulse have been initiated, the number of activations of cells at the last row indicating an arrhythmic situation should be above 2*n*, where *n* is the 2D matrix size. The explanation of why these numbers constitutes the sign of an arrhythmia is as follows. We count the total number of activations of cells in the last row. Consider the case of both initiations. In a homogeneous non-arrhythmic tissue, the number of activations of cells at the last row would be exactly 2*n*, one *n* comes from the row of "1's" (the sinus wave), while the second *n* is due to the ectopic "1" which develops into a whole 'V-shaped' wave (see Fig. 1a) appearing, non-simultaneously, as *n* operating cells at the last row. Now assume that a new driver (either a focal source or a spiral), which is experimentally detected by a phase singularity, appears in the tissue. This driver will be a source of additional arrhythmic waves which would propagate through the tissue and reach its other side. So, for the case of both initiations, if the number of activations of cells at the last row is above 2*n*, it means that additional (arrhythmic) pulses were generated in the 2D matrix implying a new driver. The case of an arrhythmia developed by a sinus initiation alone is similar to the case of both initiations but with only one n.

The case of an arrhythmia developed by a sinus initiation alone is similar to the case of both initiations but with only one *n*.

*Heterogeneity of cells in their refractory period* is shown here by cells having two kinds of periods, short and long ones. The "short" refractory period cells (termed A-type cells here) take the sequence of *1 → 2 → 3 → 0* in consecutive generations following an AP ("*1*"), while the "long" period ones (termed B-type here) take the sequence: *1 → 2 → 3 → 4 → 5 → 0*. We assume here that these B-type cells represent the *normal* heart cells, and the A-type ones depict malfunctioning short-period cells. Recall that *1* denotes an operating (i.e., AP carrying) cell, while the other numbers, besides *0* which is a waiting cell, stand for cells in their refractory period. Tissue heterogeneity is achieved by changing a percentage of the general long—period cells in a tissue into short—period ones, and the latter are distributed randomly in the 2D matrix.

### Electric conduction heterogeneity (intermittency)

We first present our postulation of the method by which cells can become intermittent and lower their conductivity (see more elaboration of this theme in the discussion section) and then study how a heterogeneity of this category can lead to arrhythmic behavior. As described in the model section, conductivity here is simulated by the influence of neighboring cells to stimulate a cell to operate.

#### Reduction of electric conduction of a single cell

When a cell is impaired (by a scar, aging, ablation), the usual "fibrosis assumption" is that it is replaced by a different type of a non-conductive (completely insulating) cell. But what if it is only slightly damaged and is not replaced? We assume that in this situation there are three possibilities: 1. Receiving intermittency: the cell's ability to reach an AP by a stimulation of an external trigger is damaged, i.e., the trigger does not always pass threshold; 2. Transfer intermittency: the cell's own triggering ability to stimulate other cells is reduced and as a result neighboring cells have a diminished probability to form an AP; 3. Combined intermittency: this type of a cell acts intermittently both in receiving and in controlling other cells' APs.

In order to simulate in our model a cell whose electric conduction is reduced in these ways, we formulated the following outline:

A cell that has a diminished electric conduction is called a "cell of *type* C". This cell can assume the usual modes of all other cells, namely 0, 1. 2… But, regarding the AP passage to and from such a cell, our design is as follows:

1. Receiving intermittency—Cell of *type* C_1_: If at the present generation any neighboring cell to a C_1_ is active (in mode 1), then, in the next generation, C_1_ becomes active only with a probability *r* (*r* < 1).
2. Transfer intermittency—Cell of *type* C_2_: If a cell of any type at the present generation, has no active cells of other types, but has at least one neighboring active cell of type C_2_, then this cell in the next generation becomes active only with a probability *r* (*r* < 1).
3. A cell having both shortages is labeled CTC.

## Results

We consider each heterogeneity in detail.

### Fibrosis

Numerous trials (not shown) were run, including distributed fibrosis of many percentages of the total cell numbers, both over the whole 2D matrix and over an *l* × *l* square within it, *l* < *n*, chosen randomly in the *n* × *n* matrix (distributed patch). The sinus node operation was simulated, as always, by a row of "1's" at the first row and the ectopic "trigger" was applied by a "1" either at the middle of the first row or at the middle of the first column or at several other positions, and at different generation-delays with respect to the row of "1's". *In no case* was the number of activations of cells at the last row above 2*n*, where *n* is the matrix size—no arrhythmia. At any rate, *no rotor or a focal*-waves* source has ever been developed*.

### Refractory period (action potential duration, APD) heterogeneity

Results obtained by our model for this heterogeneity were intended mainly to check that the main network processes were in agreement to experimental and other mathematical models' outcomes and to elucidate the influence of "obstacles" in the form of A-cells in spirals or target waves sources development. The agreements turned out, indeed, to be very good in support of our approach. In more detail: no AF was detected in refractory period heterogeneity tissues (not shown) under sinus node operation alone. Moreover, it is experimentally known that in order that a triggering ectopic pulse can generate AF, it should lag behind the sinus node wave (SNW) by an exact number of generations (vulnerable window). Coming too soon it will encounter a barrier of cells in their refractory period and would not be able to continue. Coming too late, it can spread as a target wave and disappear on the other tissue side. For a homogeneous tissue, even an additional ectopic pulse, arriving at any time in relation to the SNW, would not lead to AF. All these established processes were obtained by our model (not shown).

**Figure 2:**
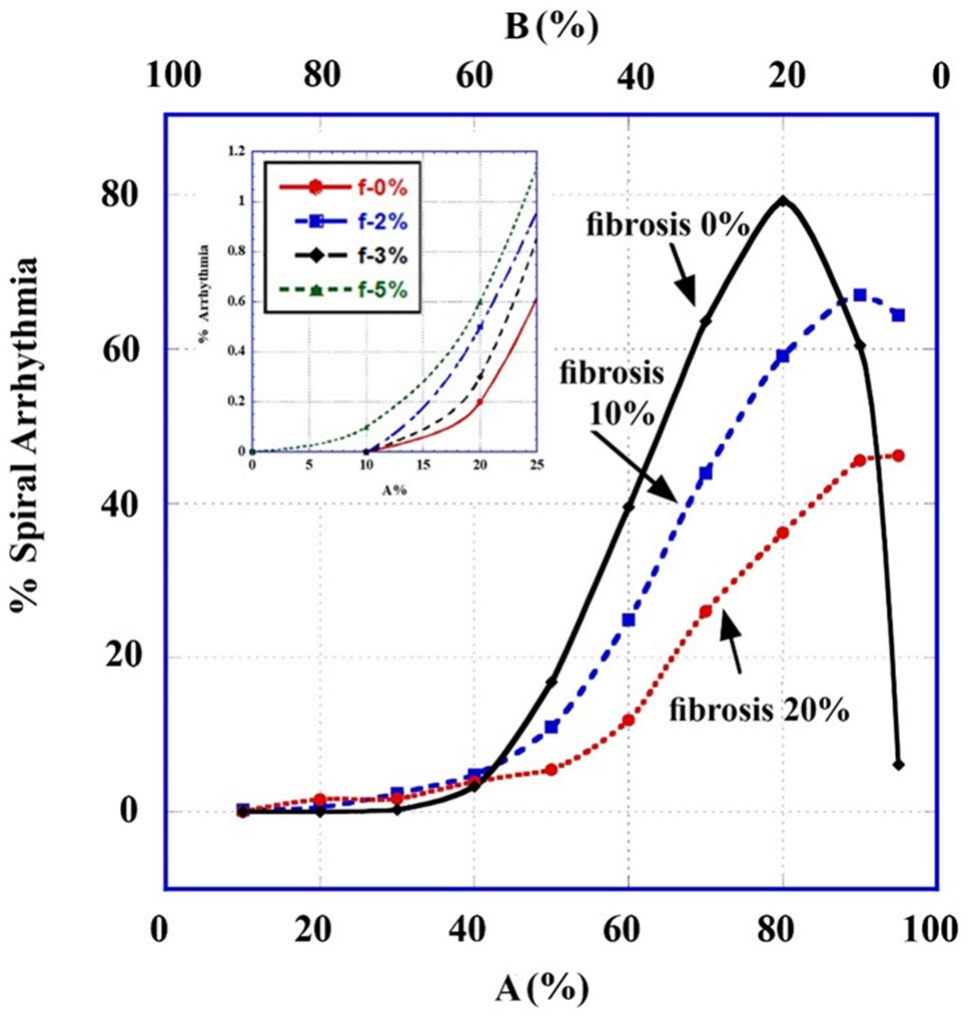
Combined refractory period and fibrosis heterogeneity arrhythmogenic statistics. The percentage of 2D (30 × 30) matrices developing AF out of 300 different randomly chosen ones are shown as a function of the percentage of A-type cells (the rest are B-type, normal, cells) in the tissue (matrix). Shown are results for 0%, 10% and 20% fibrosis. Inset: Fraction of AF for small values of A-type cells percentages.

We display here two outcomes of our model (Figs. 1, 2). In Fig. 1 is shown what happens when there exist both a heterogeneity in the refractory period and an ectopic pulse arriving at the right delay (here 4 generations behind) with respect to the SNW while in Fig. 2 is shown what happens when fibrosis is added to this heterogeneity. The 2D matrix dimensions used were 20 × 20 and 30 × 30 respectively.

The general development of an AF is as follows. The additional pulse propagates as a V-shaped target wave, its leading edge closest to the SNW (the tip of the wave) can sooner or later meet on its path an obstacle, here a short- period cell. When such an encounter occurs, this leading edge cannot continue directly on its route and the wave has to split, thus creating two "free edges". Those free edges can start building either a spiral (rotor), a double spiral (a figure-of-eight as it is called in cardiology), or an ectopic source of "target" (focal) waves. Another possibility is that there exists a unidirectional block, where the AP encounters on its one side, cells in their refractory periods and on the other side cells which have completed this period and are in their "0" state. If there are no other obstacles, a spiral may arise.

Figure 1 depicts these two possibilities (Fig. 1a—the ectopic one and Fig. 1b—the spiral one). Since our purpose is merely to understand the process, only the most important "generations" of the 2D matrices are shown.

In Fig. 1a (a finite ectopic source) is seen how the free ends created by the interaction with an A-type cell manage to create 3 consecutive target waves (waves propagating outwards): One of the free ends succeeds in driving a pulse, i.e., a cell in mode 1, retrogradely, creating a second target wave. There being not enough obstacles, the formation only "succeeds" to create just an additional scenario for a wave. These three almost complete target waves continue to move forward bringing twice the number of APs to the other side of the tissue.

Figure 1b (spiral). Here the AP finds an obstacle hindering it from going to the left in the next generation, thus starting the formation of a spiral. An almost complete spiral is developed. Since the tissue is still heterogeneous, in the following generations these patterns can break down into multiple sub-patterns resulting in fibrillation forms which can be inferred from the numbers of activations of cells at the 2D matrix final row (and see also Fig. 2). Both rotors, figure-of-eight's and "focal waves", as wave generators, can continue indefinitely, interchange, fibrillate and even terminate, if the conditions (exact tissue heterogeneity) dictate such developments.

Figure 2 portrays the statistics of the case of a combined refractory period and fibrosis heterogeneities: 300 different examples of randomly distributed heterogeneities were run for each percentage of A-type cells out of the total number of a 2D matrix cells. Shown is the total percentage of trials (arrhythmic, or AF, percentage) in which the number of activations of cells at the final destination (last row of the matrix) was larger than 60, which is the number expected by both the SNW (30) and by the V- shaped wave (30). The extra active cells are created by the additional drivers. Several interesting consequences can be derived from Fig. 2: Tissues of up to ~ 35% short-period (A type) cells in them, show no arrhythmogenic behavior. The most vulnerable region of short-period cells percentages is between 60 and 90% of the total number; Above ~ 90% of short-period cells, most of the sources die out following a certain time.

For a 20 × 20 matrix size (not shown), very similar results to Fig. 2 were obtained. Statistics of several matrix sizes (not shown) for 55% of A-type cells were checked and all showed similar statistical results of AF development.

### Electric conduction heterogeneity (a better name: Electric conduction intermittency heterogeneity, ECIH)

We examined two extreme cases.

1. When some cells still retain, say 5%, of their ability (*r* = 5%) to accept AP (C_1_) or deliver AP (C_2_) or to do both (CTC).
2. When the damage incurred to the cells is minimal and they retain, say 95%, of their abilities (r = 95%) thus evading being replaced.

**Figure 3:**
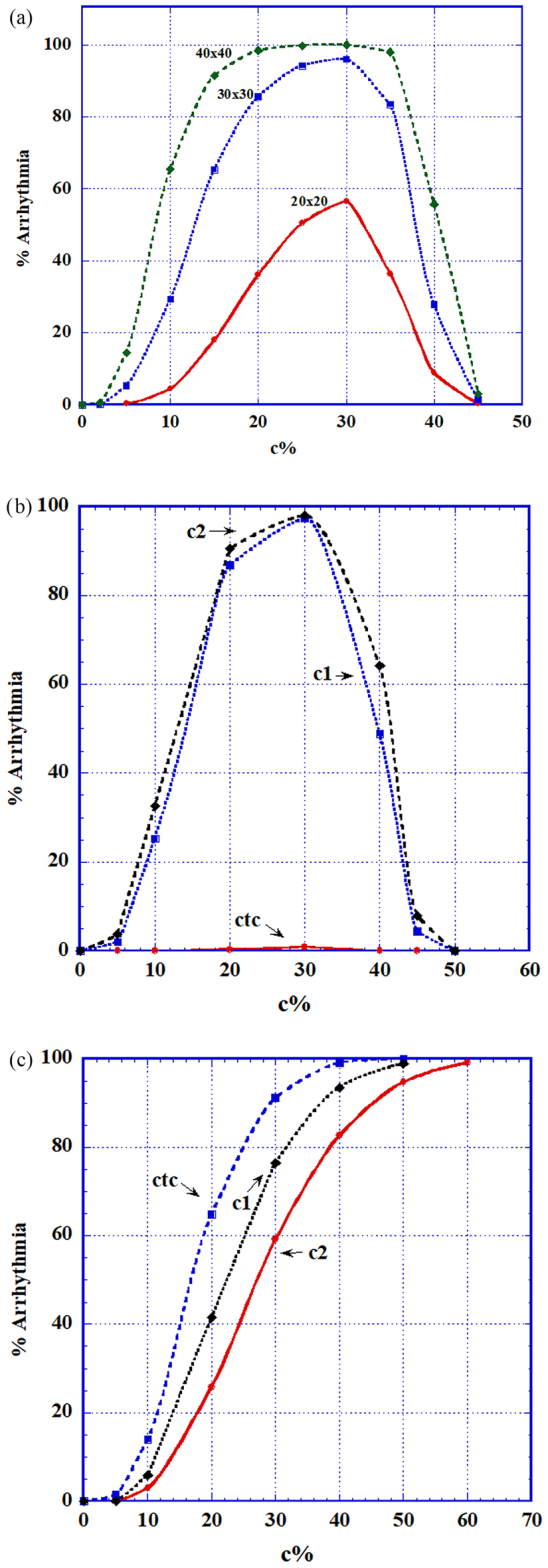
Intermittency heterogeneity arrhythmogenic statistic: The percentage of 2D matrices developing AF out of 300 different randomly chosen ones, as a function of the C-types (C1, C2 and CTC) percentage of cells in the 2D matrix where the rest of the cells are B-type, normal, cells. (**a**) Specific dependence of AF fraction on matrix size as a function of percentages of minimally competent C1 type cells: with r = only 5%. Matrix sizes shown are 20 × 20, 30 × 30 and 40 × 40. The matrix size chosen in 3b and 3c is 30 × 30. (**b**) Statistics for all minimally competent (r = 5%) C types cells; It is seen that C1 and C2 types cells behave similarly while CTC type cells perform quite differently. (**c**) Statistics for all slightly damaged (r = 95%) C types cells. AF generation is seen to increase sharply reaching 100% for all types.

**Figure 4:**
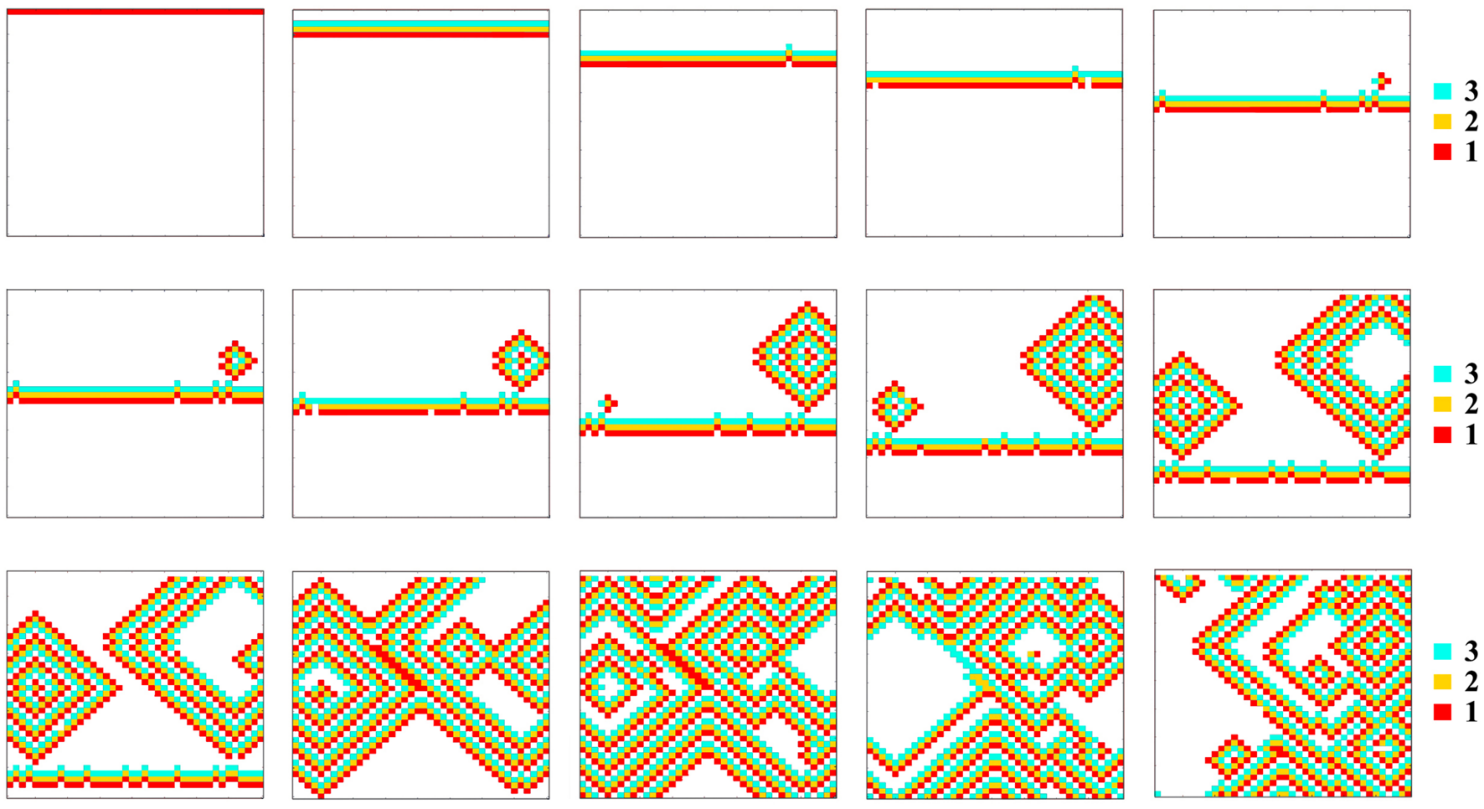
Time sequence of a combination of A- type and C-type cells in 2D matrices. Generation of a double spiral.

Heterogeneity: these r = 5% (and r = 95%) cells were inserted into tissues of completely B-type cells (*normal* or long refractory period cells) and of completely A-type cells (short refractory period) respectively. The combination of B-type and C-type cells (B + C) proved to be only relatively arrhythmogenic, while the combination of A-type and C-type cells (A + C) proved to be extremely arrhythmogenic. We display (Fig. 3) the (B + C) statistics for the three C -type cells and only show (Fig. 4) a case of double spiral generation for an (A + C) case.

Figure 3 (B + C) describes the statistics of replacing a percentage of B-type (normal) cells in a 2D matrix by C-type ones. Figure 3a displays the influence of matrix size on this kind of AF. Only the C1 case is shown but the situation is similar for the other cases. It is seen that for relatively small sizes (20 × 20 or less), size influence on the arrhythmic behavior is very strong, while for higher size values, the behavior for different sizes coalesces. Henceforth we used 2D matrix sizes $\ge 30 \times 30.$ Arrhythmic statistics in Fig. 3b demonstrates that for *r* = 5%, there is a vast difference between the behaviors of either C1 or C2 cells and the behavior of CTC cells (see Discussion).

Figure 4 shows how a combined intermittency and short QT heterogeneities can lead to a double spiral (figure of eight) under a simple sinus node operation: The line of "1's" proceeds downwards. It encounters a C-cell on its route, which, with a probability of (100-*r*) %, does not excite (remains *0* in the model). At the next generation the line of "1's" moves down one line and this *0*- cell in the previous line can (with a probability *r*) turn into an active cell (its label turning to "1"). If this happens, then the line of "1 s" will keep moving downwards with a "dent", made of a single "1", moving with it at its upper row. If, on the other hand, this cell fails to excite, then in the next generation there appears a row of two *0*'s. This row of two 0's allows a "1" to move upwards (retrograde). This "1" can either create a target wave, or, as is the situation here, the two cells on both the left and above the C-cell are also of the same type. In this case, in the next generation an open structure of three "1's" can be created (as it does here) and lead to a double spiral. Similar developments, although less frequently, arise in the B + C case.

Although not shown, many runs were calculated for the (A + C) case. It turned out that, in this highly arrhythmogenic situation, the sinus node wave can, by itself, lead to AF; there is no need for an additional trigger. Even for very low *r* values and for reduced conductivity fraction values there is a small probability to generate both ectopic and spiral sources. For example, even for *r* = 2% there are exceptional "spiral" developing events and also some "ectopic" source developments at C-type cells fractions of 18, 27 and 33%. For *r* = 5%, spirals can occur in up to ~ 30% of all runs at C-type cells fractions of 30% and also create many more ectopic sources. For *r* = 10%, the resulting spiral's percentage can reach up to 100% if there are enough "infected" cells. Higher values of *r* cause more havoc already at small fraction values.

### Combined heterogeneities

Four combinations of heterogeneities are considered: fibrosis and refractory period, fibrosis and ECIH, refractory period and ECIH and a combination of all three. Statistics are again performed by runs of 300 matrices where the average percentages of the arrhythmic ones are shown. Regarding combinations with fibrosis, recall that in our model, fibrosis by itself, while leading to delays and spreads in action potential propagation^18^, does not result in AF. Therefore, we wanted to check the potential supporting role of fibrosis to other proarrhythmic drivers. It turned out that in some cases (see below), fibrosis, by providing locations where AP sources can arise and by the above-mentioned delays and spreads, can *assist* AF creations. In different cases (see below), due possibly to its ability to hinder electric current propagation, fibrosis can *reduce* AF occurrence.

#### Refractory period and fibrosis

Results show that in combination with refractory period heterogeneity both the stopping powers and the aiding powers of fibrotic cells emerge. Thus, Fig. 2 presents the changes of arrhythmogenesis with the addition of 10% and 20% fibrosis. For low values (< 10%, see insert) and for very high values (> 90%) of the short refractory period (A-type) cells in the 2D matrix, fibrosis *enhances* AF, while for other values of these cells' numbers, fibrosis *reduces* AF. Very high values of fibrosis (> 25%, not shown) obviously lead to a drastic slowdown of AP movement, a reduction of AF and, at higher fibrosis percentages, to a shutdown of total AP transport.

#### ECIH and fibrosis

**Figure 5:**
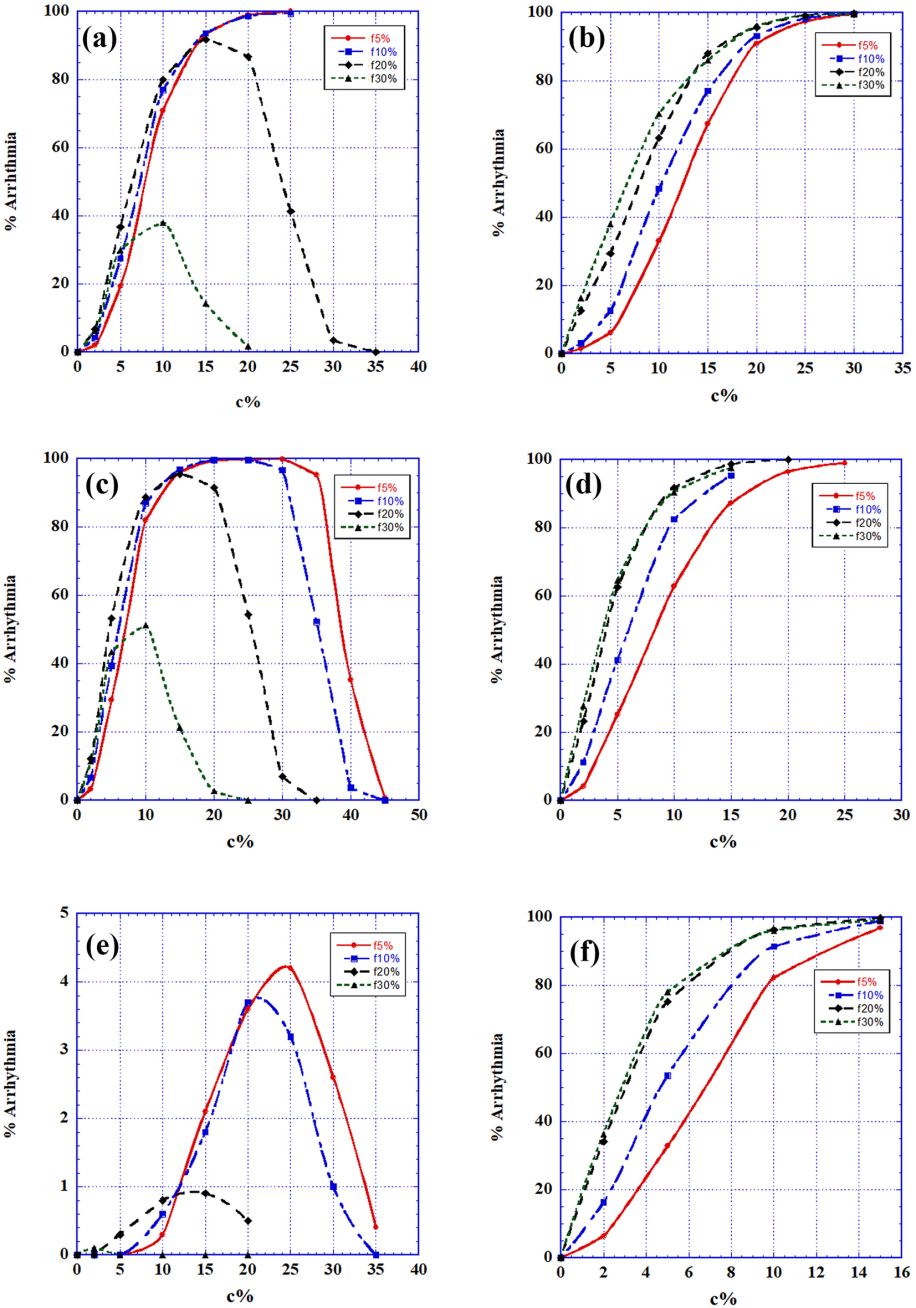
AF generation statistics of combined heterogeneity: Joint intermittency and fibrosis of strengths between 5 and 30%. (**a**) AF generation percentage as a function of minimally competent C1 type cells (r = 5%). (**b**) AF generation percentage as a function of slightly damaged C1 type cells (r = 95%). (**c**) AF generation percentage as a function of minimally competent C2 type cells (r = 5%). (**d**) AF generation fraction as a function of slightly damaged C2 type cells (r = 95%). (**e**) AF generation percentage as a function of minimally competent CTC type cells (r = 5%). (**f**) AF generation fraction as a function of slightly damaged CTC type cells (r = 95%).

We present here only a few cases. The tissue (matrix) is prepared in such a way that the percentage of C-type cells, of all types, (with *r* = 5% and r = 95%) out of the total 2D matrix-cells is changing and the remainder of the cells are of B-type (normal). Then 10% and 20% (respectively) out of the total 2D matrix-cells are replaced by fibrotic, nonconductive, cells. The final system is activated under sinus node operation and no additional trigger. Results appear in Fig. 5a,c,e—for r = 5% and for C1, C2 and CTC respectively, and in Fig. 5b,d,f—for *r* = 95% and for C1, C2 and CTC respectively.

Figure 5 shows the intricate results of the combined effects of intermittency and fibrosis:

1. Minimally competent cells (r = 5%): Without fibrosis (Fig. 3b), cells of C1 and C2 types, even when only 5% active, can induce substantial disturbance, while CTC type cells show only negligible ability to cause harm. Addition of fibrosis to small percentages of C1 and C_2_ (and even CTC) types and *r* = 5% cells, shown in Fig. 5a,c,e, displays an increase of arrhythmic capacity with fibrosis amount, even up to 30% fibrosis, while for tissues with higher percentages of the C cells, there is a reverse effect–additional fibrosis decreases AF.
2. Slightly damaged cells (r = 95%) presented in Fig. 5b,d,f: Results are shown only for rather small values of C-type percentages and display increased arrhythmogenic activity with fibrosis percentages up to 30%, but with a gradual decrease of this activity with fibrosis percentage.

#### Refractory period and ECIH

**Figure 6:**
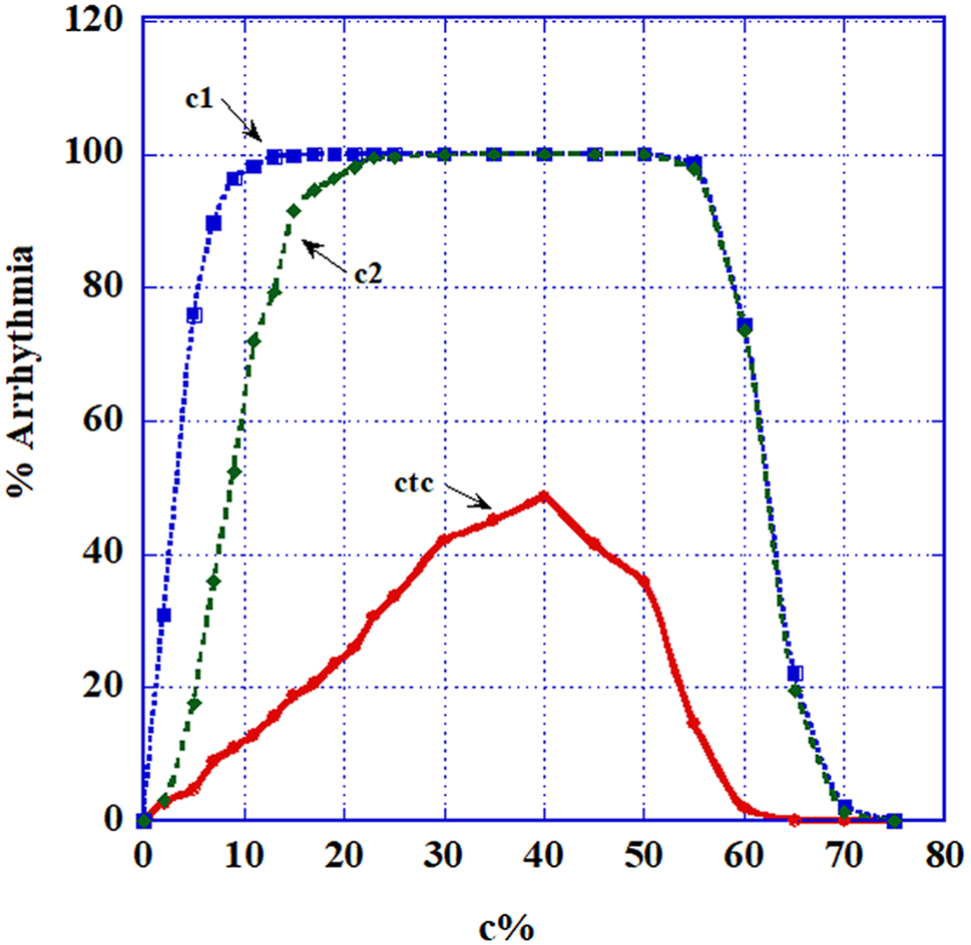
Combined refractory period and ECIH heterogeneities: 30% A-type cells; AF% as a function of changing C-types cells percentage; the other cells are of B-type. Note the synergetic increased arrhythmogenic effect.

We present here only a single case. Figure 6 depicts the arrhythmogenic percentage of 30% A-type (short refractory period) as a function of C-type (r = 5%) cells in the 2D matrix. The refractory period and ECIH heterogeneity combination turns out to be very harmful. Thus, an addition of even ~ 3% C1-type cells induces already ~ 50% probability of AF. C2-type cells also contribute a similar probability, although at somewhat less extent. And even CTC-type cells increase their disorder-promoting effect under such A-type cells addition.

#### Combined Fibrosis, refractory period and ECIH

**Figure 7:**
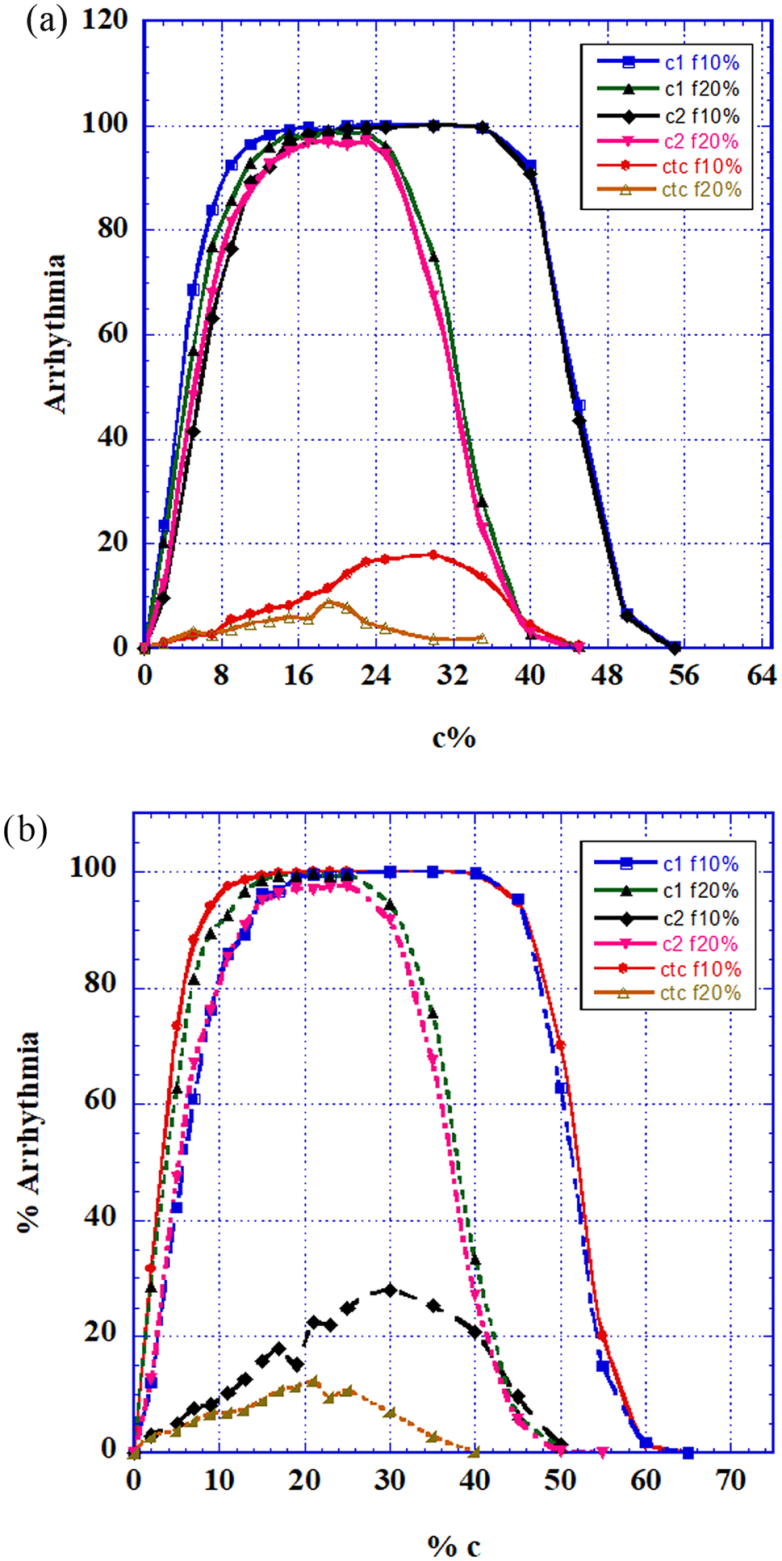
The combined effect of the three (fibrosis, refractory period and ECIH) heterogeneities: AF generation percentage as a function of changing C-types cells percentage; the other cells are of B-type (normal). (**a**) 20% A-type cells and fibrosis of 10% and of 20%. AF generation percentage as a function of percentages of the three C cell-types. (**b**) 30% A-type cells and fibrosis of 10% and of 20%.

Obviously, in clinical circumstances, such an occurrence, where all arrhythmogenic problems are combined in one heart, seems rather rare. Therefore, only 30% of the total of A-type cells were considered with different percentages (10% and 20%) of fibrotic and changing percentages of C-type (ECIH) cells (Fig. 7).

It is seen that, in such cases, fibrosis decreases arrhythmogenesis. Thus, there is a high decrease of this malfunction probability in Fig. 7 with respect to Fig. 6, both for relatively small and for relatively large C-type percentages.

## Discussion

In summary, results obtained for the different heterogeneities (substrates) indicate the following features:

### Fibrosis

Distributed fibrosis in our model is the "ultimate" heterogeneity of electric conductivity since fibrotic cells are *completely* insulating while the other cells are conducting (at least to some degree). It turned out that such distributed fibrosis, both when it is spread throughout the whole tissue or when it is constrained to a restricted portion of it, and when no other heterogeneities are present, *did not lead to AF* in the present model, even when both contributing conditions were present, the heterogeneity and the ectopic trigger. This result means that, at least for the present model, distributed fibrosis per se is not a true AF generating condition and can possibly only aid (or hinder) other proarrhythmic agents (see below). Other studies^28,29^ have shown that hindrance of propagating waves can a) promote wave breaks and b) slow the conduction velocity sufficiently to sustain re-entrant circuits for a long time / indefinitely. Both factors are, of course, proarrhythmic. In our model such wave breaks were not obtained.

Since our model is rather simple and is looking at quite small tissue sizes, and since in more sophisticated models with fibrosis on tissue sizes of 500 × 500 or even 1000 × 1000 cells it was shown that fibrosis is arrhythmogenic, our inferences might disagree with those results.

### Refractory period

Our results, that are in agreement with the known experimental ones^29^, specifically show that "unaided" refractory period heterogeneity, implying short action potential duration (APD) cells, does not lead to AF under sinus rhythm alone. An additional ectopic trigger at the right time is needed for such malfunction to occur. Results also indicate that:

1. Under the action of both heterogeneity and an ectopic AP pulse, the necessary and sufficient conditions for an arrhythmic behavior are established.
2. The proarrhythmic influence of a short refractory period of even a small number of cells is significant.
3. Even under these necessary and sufficient conditions, AF occurrence is not certain, its probability depends on the heterogeneity percentage and on the delay (vulnerable window) between the sinus node wave and the appearance of the ectopic pulse(s).
4. Two types of AF drivers can arise under these conditions, a less abundant "target" waves (focal) source and a spiral source. Both depend on the ratio of short to long refractory periods. The most "hazardous" long to short ratio is below 3:1, where the AF generation probability is almost 100%.

### Electric conduction heterogeneity (ECIH)

We considered the arrhythmic danger of such C1, C2 and CTC cells with either very large or very small *r* values. Results show that *cells having these extreme r values are capable of producing AF in significant percentages of trials, even under sinus node function alone*. Specifically (Fig. 3): the C1 and C2 types show similar behavior rising even up to ~ 95% arrhythmia probability between ~ 20% and ~ 35% of their tissue-presence, while the CTC case does not show any development of this sort. This unexpected null result is possibly due to the fact that, by decreasing both their inputs and outputs to 5%, these cells become almost completely nonconductive and as such almost belong to the fibrotic group. For *r* = 95% on the other hand, no such difference is observed. Rather, it turns out that the presence, above a certain percentage (~ 15%) of the total cell number, of C-type cells of such a mild intermittency can lead to severe (up to 100%) probability for AF. But note that low concentrations of C1 and C2 cells of r = 5% are much more arrhythmic than cells of these types with r = 95%. These statistics were calculated under both a sinus node wave and an additional ectopic pulse. Similar statistics were obtained (not shown) for runs under a *sinus wave alone*, with no additional triggering.

The ECIH problem turns out to be very size dependent. Figure 3a shows that for matrices, each of which contains the same percentage of intermittent cells, AF increases with matrices' size. Thus, unlike the refractory period heterogeneity, the severity of ECIH "effectiveness" depends on the size of the heart's area inflicted by this disfunction. The larger this area is, the higher is the AF probability.

ECIH in our model turned out to be the *most proarrhythmic system*. Even a small number of such ECIH-cells and with *r* values close to 0 and slightly below100% can cause AF.

### Combined heterogeneities and fibrosis

In combination with other arrhythmogenic agents, fibrosis can perform *dual* tasks. In conjunction with both refractory period and ECIH heterogeneities it constitutes a *very efficient assisting agent* of AF for low arrhythmogenic cell concentrations of either type, while *reducing* arrhythmogenic initiation for higher concentrations. The latter behavior can possibly be explained if we realize that fibrosis, by its nature, can hinder arrhythmic assemblies (e.g., spirals) from propagating. Very severe fibrosis of course can lead to a temporary or even a complete AP blockage.

Specifically:

1. (Fig. 2). Combined with refractory period heterogeneities: for very small values of A-type cells in the tissue, fibrosis aids AF (see insert). It might be considered that for this range of A-type cells, heterogeneity is low (almost every cell is B-type) and fibrosis supports AF by enhancing heterogeneity. On the other hand, fibrosis AF obstruction facilities appear for higher values of A-type cells, resulting in the reduction of the malfunction. Evidently, part of the reduction comes from the replacement in the model of part of the A -type cells by fibrotic (non-conducting) ones.
2. (Fig. 5). Fibrosis synergetic combination with ECIH heterogeneities increases the probability of arrhythmic outcomes in the tissue for small and medium ECIH concentrations, while for high ECIH concentrations, similar to the refractory period case, it obstructs arrhythmic occurrence. It is also interesting to see the reduction of the facilitating effect of fibrosis as a function of *its* concentration, when its conductivity obstruction starts to compete with its facilitating effect.
3. (Fig. 7). Combination of all arrhythmogenic heterogeneities. The behavior, calculated for implanting 20% or 30% short APD and C-type cells in the tissue, shows only the actual health benefit of fibrosis in such extremely arrhythmogenic situation, where its presence *reduces* the malfunction probability.

### Combined refractory period and ECIH (Fig. 6)

As noted above, this combination of heterogeneities is the most arrhythmogenic one. It seems that the short refractory periodic cells can contribute obstacles, which increase the probability of a one-way temporary block in the AP propagation.

All conclusions presented henceforth are related to the present model but can represent actual heart tissue behavior.

Analyzing our results, we can deduce the following observations:

### Fibrosis

Fibrotic tissues show some perturbing behaviors^18^ such as a delay of the AP, an increased travel times across them (tortuosity), uneven temporal throughputs and, when fibrosis is too severe, a complete blockage of the AP passage.

Note that, in the extreme arrhythmic vulnerability of the tissue to "electric conduction heterogeneity" (type C), if there existed a means by which "slightly damaged" cells, i.e., cells that have a diminished electrical conduction, could be turned into fibrotic cells, i.e., complete insulators, it would improve the tissue antiarrhythmic resistance.

### Refractory period heterogeneity

In^30^ the refractory period heterogeneity is investigated in canine models. It is ascribed to tissue remodeling, and relevant drug treatment applications are analyzed. Our results agree with^30^ in that both models, the animals' and the mathematical ones, show that under the appropriate conditions, this heterogeneity can be highly arrhythmogenic.

### ECIH

We call this cellular state and heterogeneity an "electric conduction" one, but our simulation is somewhat different from the traditional approaches to electric conduction.

The two extreme cases treated here correlate with two of the issues (riddles 1 and 2 of^31^): small *r* values might correspond to the "remaining" ability of nearly dead cells, while *r* values close to 100%, could correspond to a toxin slightly-damaged persistent cell.

Let us consider our model simulation of "reduced cell conductivity". It can be argued that this kind of simulation does not truly represent such a reduction, since existing research (Ref.^32^ and references therein) ascribes reduced tissue conductivity to the existence of cells becoming *complete insulators* by scarring, fibrosis or ablation. The reduced conductivity of the tissue in this case is exhibited by the zigzag route^31^, or tortuosity, of the AP through the cellular medium. This type of conductivity reduction can be detected by a prolonged PR period in the EKG^33^. We suggest an additional possible mode of reduced electrical function of individual cells, causing thereby a partially reduced or an *intermittent* cellular conduction.

We now present some plausible processes that can lead to such an intermittency.

1. Cell death can occur in many ways by apoptosis or necrosis. In the fibrosis process, such dead cells are replaced by connective nonconductive cells. However, there may exist cells which escape death, either because of different genetic base, or simply because they are "persistent"^32^. At any rate, we assume that some "damaged" cells would survive the cellular death process and can become C-type ones, exhibiting intermittent conduction behavior characterized by *r* (see 3. below).
2. Aging may deteriorate cell functions and lead to changes of biological levels (see 4. below).
3. Drugs, for example those treating hERG (the human Ether-à-go-go-Related Gene) channels or Vaughan-Williams class I drugs, mainly sodium channel blockade ones^34^, can change the action potential *threshold* of cells. If drug delivery or the response to the drug is not homogeneous, it can lead to the alteration of this threshold in only a fraction of cells, which in turn can induce conduction intermittency in these cells.
4. Regarding the feasibility of C-type cells, let us recall that biological levels are never completely equivalent to each other. For example, in cardiology: "There are many possible sources of randomness that can be responsible for the seemingly unpredictable nature of AFs"^35^. Thermodynamic fluctuations (noise) and fluctuations in ion channel dynamics influence AP gating. These phenomena cause cell changes which can possibly lead to the malfunctions exhibited by the C-types cells:

C1: Assume that the cell-threshold for an AP pulse has increased, so that the pulse heights arriving at the cell become of approximately the height of this threshold. Since, as mentioned before, biological values (here the arriving pulse heights) are never absolutely equivalent to each other, some pulses will trigger this cell's AP, and some will fail to do so.

C2: Assume that the AP pulse-heights *emitted* by the cell have dropped, so that they are of the approximate height of the average healthy cell threshold. Then, some of this cell's emitted pulses will trigger APs in the accepting cells, while some will fail to do so.

CTC: When both C1 and C2 shortages occur simultaneously.

Bearing in mind the discrete nature of the heart (built of discrete cells), the nonlinearity of the electric conduction process there and the possibility of a malfunction in the apoptosis/necrosis process, or of the mixed influence of drugs, it seems to us that our intermittency assumption might be a representation of an actual problem in addition to the present on–off conductivity reduction.

The intermittent conduction described here is, in a sense, an opposite process to afterdepolarization (usually arising from long QT Syndrome, LQTS). In the ECIH there is a probability of *dropping* a regular AP pulse while in the afterdepolarization one there is a probability of *adding* a pulse to a regular AP one.

A somewhat different kind of "intermittency" in the heart electrical response is cardiac alternans. It is manifested as changes, either in the action potential duration or in the cytosolic calcium transient amplitude. In EKG, alternans can be detected as T-wave alternans, which mark the possibility of cardiac arrhythmias. However, the alternans' big and small alterations are *periodic* while the conduction intermittency of ECIH is assumed to be *stochastic*.

Intermittency can be a temporary occurrence, with an appearing time and a disappearing time. Here we treat the system only during the time in which intermittently is active.

One of the most deleterious complications of the cardiac tissue is a *temporary* unidirectional block^36^. This problem can lead to the development of a spiral of action potential which may induce tachycardia and, encountering obstacles, this spiral (rotor) can split into small spirals causing fibrillation. Now, a C-type cell in its nonconductive stage fulfils exactly this function, namely a block lasting only until the cell conducts again. It is therefore no wonder that such intermittent cells, if they exist, can be detrimental to the tissue.

Therefore, we posit that this kind of malfunction should not be neglected. We call for an experimental effort to detect cells of these types of intermittencies. If such cells are detected, a possible remedy might be "Engineering more conductive scars"^37^. Failing this approach, rendering these cells into being completely insulating might help.

Our results show that in some cases, arrhythmogenic ECIH-cells are *themselves* the centers of the AF, but in other cases they can induce arrhythmic centers at *different* locations than their own by driving retrograde pulses. In the cases when intermittent cells are the centers of AF, clinical detection of these C-type cells is already unintentionally carried out, although not in the "intermittent" identity. Clinically, these cells reside at the centers (the singular phases) of the structures causing the arrhythmogenic disturbance, either at the center of a rotor or at that of an ectopic source. These centers are anyhow the ones looked for in the ablation procedure (e.g., the OPTIMA ablation procedure^38^). Therefore, for clinical ablation purposes in these cases it does not matter what the nature of the malfunction leading to AF is. However, if these cells are *not* themselves such centers, mere ablation of the centers may not be enough, since the differently positioned unattended intermittent cells could lead to AF at other sites. Therefore, in such cases, for treating the problem and possibly for finding new remedies for it, ECIH understanding seems essential. In such cases identifying these C-type cells becomes an important measure to curb AF, though such detection of an intermittent cell might prove to be a difficult task. For, as is well known, intermittency detection is difficult even in electrical engineering, as can be inferred by the many approaches applied to counter this problem in this realm.

An interesting feature of C-type induced AF is its size dependence. Small "problematic" areas are much less proarrhythmic than larger ones.

In a recent paper^39^ reviewing the AF problem it was stated that "Studies on the histological as well as electrophysiological aspects of the disease have led to its better understanding, improving the therapeutic possibilities and effectively, the quality of life of patients. However, crucial questions regarding the formation and perpetuation of the disease remain unanswered." In this work we have tried to partly answer these questions.

One limitation of the present study is that we have not considered in our model the electrotonic coupling that exists within the atrial tissue. This coupling was shown^40^ to result in a reduction of variability and hence of heterogeneity impact. However, since "some diseases cause an uncoupling between cells, and therefore an increase in cellular variability is to be expected in unhealthy tissue"^40^. It means that our model studies the effects resulting after the electrotonic coupling reduction was considered.

A different form of intermittency is considered in Ref.^41^. In this study a cardiac model is used to investigate the behavior of an already existing spiral in a heterogeneous tissue model, and it is shown that, "depending on the size and strength of the heterogeneity, the spiral wave is either permanently, *intermittently*, or not trapped at all."

## Conclusion

AF, when formed in our model, is developed from a heterogeneity in the tissue by forming ectopic (less frequent), spiral (rotor) or double-spiral generators.

The main conclusion we can draw from this work in relation to heterogeneities of proarrhythmic agents (PAs) is that t*he most "efficient" PAs are electrical conduction intermittent cells*. They can lead to AF even under sinus rhythm alone (no need for an additional pulse).

Other inferences are: that refractory period heterogeneity is an effective PA, but to induce AF it needs both a sinus AP and an exactly delayed additional AP pulse that diffuse fibrosis on its own is not arrhythmogenic (note however the reservation mentioned before to this conclusion) but can act in assisting other PAs to promote AF, while in highly arrhythmogenic situations it can reduce AF; and that the combined effect of different PAs can be highly synergetic in their arrhythmogenic power.

## Data Availability

Data will be available on request from Prof. A Rabinovitch.
